# Validation of publicly-available software used in analyzing NGS data for HIV-1 drug resistance mutations and transmission networks in a Washington, DC, Cohort

**DOI:** 10.1371/journal.pone.0214820

**Published:** 2019-04-09

**Authors:** Kamwing Jair, Chase D. McCann, Harrison Reed, Amanda D. Castel, Marcos Pérez-Losada, Brittany Wilbourn, Alan E. Greenberg, Jeanne A. Jordan, the DC Cohort Executive Committee

**Affiliations:** 1 Department of Epidemiology and Biostatistics, Milken Institute School of Public Health, The George Washington University, Washington, DC, United States of America; 2 Department of Immunology and Microbial Pathogenesis, Weill Cornell Graduate School of Medical Sciences, New York, NY, United States of America; 3 Department of Forensic Sciences, Public Health Laboratory, District of Columbia, Washington, DC, United States of America; 4 GWU Computational Biology Institute and CIBIO-InBIO, Centro de Investigação em Biodiversidade e Recursos Genéticos, Universidade do Porto, Campus Agrário de Vairão, Vairão, Portugal; Consejo Superior de Investigaciones Cientificas, SPAIN

## Abstract

The DC Cohort is an ongoing longitudinal observational study of persons living with HIV. To better understand HIV-1 drug resistance and potential transmission clusters among these participants, we performed targeted, paired-end next-generation sequencing (NGS) of *protease*, *reverse transcriptase* and *integrase* amplicons. We elected to use free, publicly-available software (HyDRA Web, Stanford HIVdb and HIV-TRACE) for data analyses so that laboratory personnel without extensive bioinformatics expertise could use it; making the approach accessible and affordable for labs worldwide. With more laboratories transitioning away from Sanger-based chemistries to NGS platforms, lower frequency drug resistance mutations (DRMs) can be detected, yet their clinical relevance is uncertain. We looked at the impact choice in cutoff percentage had on number of DRMs detected and found an inverse correlation between the two. Longitudinal studies will be needed to determine whether low frequency DRMs are an early indicator of emerging resistance. We successfully validated this pipeline against a commercial pipeline, and another free, publicly-available pipeline. RT DRM results from HyDRA Web were compared to both SmartGene and PASeq Web; using the Mantel test, R^2^ values were 0.9332 (p<0.0001) and 0.9097 (p<0.0001), respectively. PR and IN DRM results from HyDRA Web were then compared with PASeq Web only; using the Mantel test, R^2^ values were 0.9993 (p<0.0001) and 0.9765 (p<0.0001), respectively. Drug resistance was highest for the NRTI drug class and lowest for the PI drug class in this cohort. RT DRM interpretation reports from this pipeline were also highly correlative compared to SmartGene pipeline; using the Spearman’s Correlation, *r*_*s*_ value was 0.97757 (p<0.0001). HIV-TRACE was used to identify potential transmission clusters to better understand potential linkages among an urban cohort of persons living with HIV; more individuals were male, of black race, with an HIV risk factor of either MSM or High-risk Heterosexual. Common DRMs existed among individuals within a cluster. In summary, we validated a comprehensive, easy-to-use and affordable NGS approach for tracking HIV-1 drug resistance and identifying potential transmission clusters within the community.

## Introduction

More laboratories are considering transitioning away from Sanger-based chemistries to Next Generation Sequencing (NGS) platforms for generating data on HIV-1 drug resistant mutations (DRMs). This switch would result in detecting minor populations of drug resistant variants well below the 15–20% frequency threshold achievable with Sanger sequencing [1]. Although official guidance is not yet available around which percentage cutoff threshold is considered clinically relevant, there are accumulating studies that associate low-frequency DRMs and treatment failure [2–6].

With the World Health Organization (WHO) recommendation to use dolutegravir as an alternative first-line HIV regimen [7], it will be important for sequencing laboratories everywhere to build capacity to screen for DRMs impacting all four drug classes including Reverse Transcriptase Inhibitors (NRTIs and NNRTIs), Protease Inhibitors (PIs) and Integrase Strand Transfer Inhibitors (INSTIs) [8].

In this paper, we describe a comprehensive approach for sequencing HIV-1 RNA from plasma using the Illumina MiSeq platform for targeted, paired-end sequencing of the *pol* gene including *protease* (PR), *reverse transcriptase* (RT) and *integrase* (IN) amplicons. Importantly, we chose to use free, publicly-available software that laboratory personnel without extensive bioinformatics expertise could use; making it both accessible and affordable for labs worldwide. We chose HyDRA Web to detect DRMs [9], the Stanford HIVdb program to generate DRM interpretation reports [10], and HIV-TRACE to assess transmission clusters [11–13]. We successfully validated this approach by comparing data generated using this pipeline to both a commercial pipeline (SmartGene IDNS 5 for HIV-1 Deep-Sequencing) [14] and another free, publicly-available Web-based pipeline (PASeq) [15].

## Materials and methods

### Description of study population and participant enrollment

Written informed consent was obtained from all participants prior to enrollment in the DC Cohort and the molecular epidemiology sub-study. The DC Cohort and molecular epidemiology studies were approved by the Institutional Review Board the The George Washington University (IRB #071029), which serves as the IRB of record for Whitman-Walker Health, La Clinica del Pueblo, Family and Medical Counseling Service, Unity Health Care, The GW Medical Faculty Associates, MetroHealth, and Children’s National Health System (pediatric and adolescent clinics). The study was independently approved by the IRBs of record at Howard University Hospital (adult and pediatric clinics), MedStar Washington Hospital Center, Georgetown University, and the Veterans Affairs Medical Center.

The DC Cohort is an ongoing longitudinal observational cohort study that began in 2011 [16]. To date, the study follows over 9,000 PLWH receiving care at one of 15 clinics within Washington, DC. In 2015, we obtained funding to develop a targeted NGS approach for assessing HIV-1 DRMs and for building transmission clusters among cohort participants. Between January 2016 and May 2017, cohort participants were approached and asked about their interest in participating in this molecular epidemiology sub-study. Written informed consent was obtained from 79 eligible cohort participants at eight clinics. The HIV diagnosis year for these 79 DC Cohort participants ranged from 1984 to 2016.

Eligibility criteria included: current DC cohort enrollment, ≥18 years of age, HIV-1 viral load (VL) ≥1,500 copies/mL, written informed consent, providing a blood sample for sequencing and completing a behavioral survey. Seventy-nine consented participants had at least 1 amplicon generated from HIV-1 PR, RT and/or IN targets. NGS data were paired with demographic and clinical data. Univariate analyses (i.e., descriptive statistics) were conducted to describe and compare 79 participants by their ARV treatment status.

### Sample collection and storage

Seventy-nine eligible and consented cohort participants provided two 8 mL-K_2_EDTA-containing tubes of blood (BD, Oakville, ON) for this study. Specimens were transported on cold packs to the laboratory within an hour of venipuncture or in a few occasions were refrigerated overnight at 4°C before transport. Bloods were centrifuged 10 min at 1000 rcf, with plasma recentrifuged 10 min at 1000 rcf. Processed plasma was stored in 1 mL aliquots at -80°C within 6 hours of venipuncture.

### RNA extraction, cDNA synthesis and targeted PCR

Total RNA was extracted from 150 μL of plasma (QIAamp Viral RNA Mini Kit, Cat. #52904, Qiagen, Gaithersburg, MD) according to manufacturer’s instructions. RNA was eluted in 50 μL of elution buffer and stored at -80°C prior to cDNA synthesis. RNA quality was assessed using Bioanalyzer (RNA 6000 Pico Assay Kit, Cat. #5067–1513, Aglient, Santa Clara, CA). RNA quantity was measured using Qubit 3.0 (RNA HS Assay Kit, Cat. #Q32855, Invitrogen, Thermo-Fisher Scientific, Waltham, MA).

cDNA was generated using SuperScript IV First-Strand Synthesis System (Cat. #18091050, Invitrogen, Carlsbad, CA). Briefly, 6 μL RNA were mixed with 1 μL random hexamer primers (50ng/μL), 1 μL dNTP mix (10mM) and 5 μL nuclease-free water followed by incubation with SuperScript IV master mix according to manufacturer’s instructions.

Primers (Table 1) were designed to target HIV-1 PR, RT and IN regions informative for DRMs. To avoid primer competition, each target was amplified individually (SYBR Premix Ex Taq master mix, Cat. #RR420A, Takara Bio USA, Mountain View, CA). We designed multiple primer pairs for each gene target to increase chances of generating amplicons for sequencing.

**Table 1:** HIV-1 PCR primers.

Set	Primer	Sequence (5' - 3')	Position (nt)^a^	Annealing Temperature (°C)	Product size (nt)^a^
**Protease**					
1	HIV-1_PR-2F	CACCAAATGAAAGATTGTACTGAG	2050–2073	2F/2R: 56	678
	HIV-1_PR-2R	CTGGAGTATTGTATGGATTTTCAGG	2703–2727		
2	HIV-1_PR-4F	GAGGCAATTTTAGGAACCAAAG	1928–1949	4F/4R: 55	883
	HIV-1_PR-4R	GAAGTCTTGAGTTCTCTTATTAAGTT	2785–2810		
**Reverse Transcriptase**					
1	HIV-1_RT-1F	TAAAAGCATTAGTAGAAATTTGTAC	2641–2665	1F/1R: 56	622
	HIV-1_RT-1R	TATCAGGATGGAGTTCATAACC	3240–3261		
2	HIV-1_RT-2F	GTTAAACAATGGCCATTGACAG	2610–2632	2F/1R: 56	652
	HIV-1_RT-1R	TATCAGGATGGAGTTCATAACC	3240–3261		
**Integrase**					
1	HIV-1_IN-2F	GTGCTGGAATCAGGAAAGTACT	4207–4228	2F/2R: 53	967
	HIV-1_IN-2R	CATAGTGATGTCTATAAAACCATCC	5149–5173		
2	HIV-1_IN-5F	GGAATTGGAGGAAATGAACAAGTAG	4170–4194	5F/5R: 53	997
	HIV-1_IN-5R	ATGTCTATAAAACCATCCCCTAGC	5143–5166		
3	HIV-1_IN-3771F	GCCACCTGGATTCCTGAGTG	3771–3790	3771F/5266R: 53	1496
	HIV-1_IN-5266R	CTCCTGTATGCAGACCCCAATAT	5244–5266		
4	HIV-1_IN-4074F	CAACCAGATAAAAGTGAATCAG	4074–4095	4074F/5266R: 53	1193
	HIV-1_IN-5266R	CTCCTGTATGCAGACCCCAATAT	5244–5266		

^a^ According to HIV-1 reference strain HXB2 (GeneBank accession number K03455). nt, Nucleotides.

All amplicons from a participant visible on 1% agarose gel (Cat. #BP164-100, Fisher Scientific) were pooled together and purified using Agencourt AMPure XP beads (Cat. #A63880, Beckman Coulter, Brea, CA) according to manufacturer’s instructions. Nucleic acid concentrations were measured using Qubit dsDNA HS Assay Kit (Cat. #Q32854, Invitrogen) prior to library preparation.

### Library preparation and NGS

Participant’s pooled amplicons were fragmented, and adapter sequences added for indexing (Nextera XT Library Prep kit, Cat. #15032350 and XT Index Kit Cat. #15055294, Illumina, San Diego, CA) according to manufacturer’s instructions. After a final purification step using Agencourt AMPure XP beads, resulting libraries were assessed for quality (Bioanalyzer) and quantity (Qubit). Samples (4 nM) with different indexes were pooled, loaded onto Illumina MiSeq platform at 10 pM final concentration and sequenced using MiSeq v2 (300 cycles) kit (Cat. #MS-102-2002, Illumina) according to manufacturer’s instructions. To increase diversity, PhiX Control v3 DNA (Cat. #15017666, Illumina) was added at 10% to the indexed library pool prior to sequencing.

### NGS quality control prior to data analyses

CLC Genomics Workbench version 10.0 (Qiagen) was used to quality control raw paired-end reads and to map each fastq file to HIV-1 reference HXB2 (Accession #K03455). Number of mapped reads, mapping percentage, read length and track graphs from each amplicon were examined for quality prior to analyzing data. Only those sequences exceeding the baseline threshold ≥Q30 score for quality were included in the data analyses. The SRA accession number for this submission is PRJNA517147.

### HyDRA web application used as NGS pipeline for detecting four classes of DRMs

**Fig 1:**
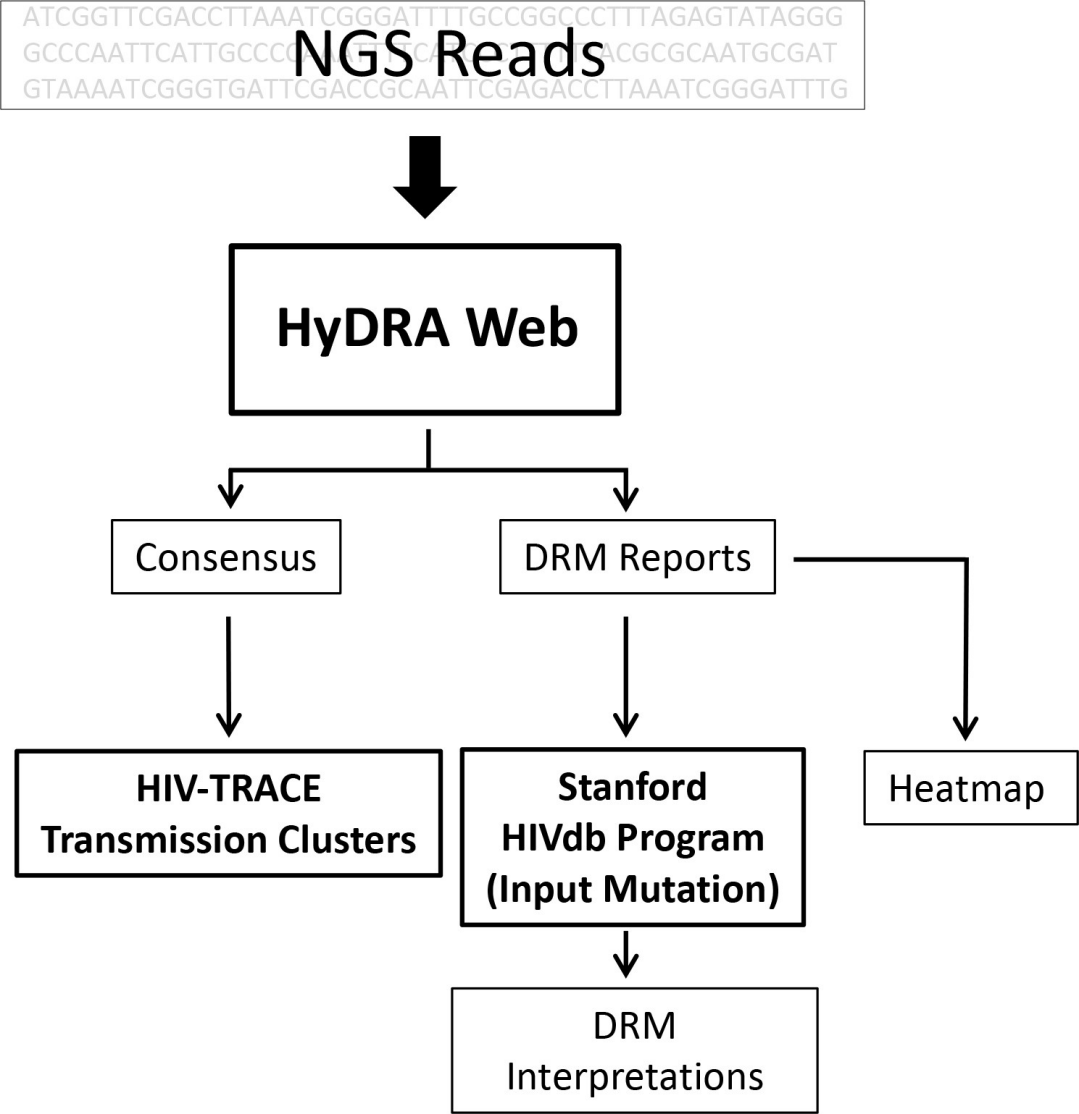
Bioinformatic pipeline used to analyze NGS sequences from PR, RT and IN targeted amplicons. Bolded boxes represent publicly-available software used in analyses. Consensus sequences, generated using a ≥20% frequency cutoff, were used in HIV-TRACE to generate transmission clusters, while DRM Heatmaps and Interpretation reports were generated using sequences at a ≥1% frequency cutoff.

Fig 1 illustrates the pipeline workflow we chose to analyze NGS data. Paired-end reads (fastq) from Illumina MiSeq were uploaded into the HyDRA Web application v1.2.5-172e7c6 (https://hydra.canada.ca/) for analysis [9]. Minimum amino acid (a.a.) frequency for DRM variant calls was set at 1% cutoff. The impact choice in frequency cutoff (>20%, >15%, >10%, >5% and >1%) had on number of DRMs detected across the four drug classes including Protease Inhibitors (PIs), Nucleoside Reverse Transcriptase Inhibitors (NRTIs), Non-Nucleoside Reverse Transcriptase Inhibitors (NNRTIs) and Integrase Strand Transfer Inhibitors (INSTIs) was also assessed. We chose to look at percentage cutoffs from 20% to 1% because the upper end (15–20%) reflects what can be detected using Sanger-based platforms, while the lower end (1–5%) reflects what is possible using NGS platforms. Default settings were applied for all other analysis parameters.

### HyDRA web application validated against other web-based applications for DRM detection

The DRMs detected from 78 RT sequences using HyDRA Web were compared to those obtained using Web-based SmartGene IDNS 5 for HIV-1 Deep-Sequencing (SmartGene, GmbH, Lausanne, Switzerland) (www.smartgene.com) and to the Web-based PASeq (www.PASeq.org). DRM frequency data was illustrated as Heatmaps using Microsoft Excel. Conditional formatting function was applied to generate a color gradient scale from white (0% DRM) to darkest color (100% DRM). The Mantel test was used to determine correlations between HyDRA Web and SmartGene matrices, as well as between HyDRA Web and PASeq Web matrices. The R^2^ and p-values were calculated from these correlations.

The DRMs detected from the PR and IN sequences using HyDRA Web were compared only to those obtained using PASeq Web. The Mantel test was again used to determine correlations between HyDRA Web and PASeq Web matrices, with R^2^ and p-values calculated.

### Stanford HIVdb RT DRM interpretation reports validated against SmartGene IDNS 5 for HIV-1 Deep-Sequencing

The RT drug resistance mutations detected using HyDRA Web were manually entered (input by mutation) and queried using Stanford Drug Resistance Database (HIVdb) Version 8.5 (https://hivdb.stanford.edu/hivdb/by-mutations) [10]. Interpretations from these DRM reports were generated for each participant for 7 NRTIs and 4 NNRTIs and these data compared to those generated using SmartGene. Both the HyDRA Web and Stanford HIVdb pipelines use the same 5 interpretation categories (Susceptible, Potential Low-Level, Low-Level, Intermediate and High-Level Resistance) for their analyses. To be considered a perfect match, interpretations from the 2 pipelines needed to be identical. The Spearman Correlation was used to calculate *r*_*s*_ to measure the strength of association between the DRM interpretations.

### Transmission clusters generated using HIV-TRACE

The HIV-TRACE platform (test.datamonkey.org/hivtrace) was used to build potential transmission clusters. The data used in the analyses consisted of HIV-1 RT consensus sequences and demographic data regarding sex/gender, race/ethnicity and HIV behavioral risk factors [11–13]. Parameters included: Cutoff for generating consensus sequences (20%), Minimum overlap (500 bp), Distance Threshold (0.015), Ambiguity Handling (Resolve) and Ambiguity Fraction (0.015).

## Results

### Description of DC cohort participant characteristics and laboratory results

Table 2 summarizes participant demographic characteristics. Among the 79 participants more were male, Non-Hispanic Black and reported an HIV risk behavior category of either high-risk heterosexual contact (HRH), or were men who had sex with other men (MSM). Statistical differences were found when comparing the 11 treatment-naïve to the 68 treatment-experienced participants and included median age, median year of HIV diagnosis, race and CD4 counts (both nadir and current median counts). The most common HIV-1 subtype seen among this sub-set of DC Cohort participants was B (88.3%), followed by F1 (9.3%), C (1.2%) and CRF02_AG (1.2%). Of the 79 participants, 66 (83.5%) reported residing in DC, 9 (11.4%) in MD, 3 (3.8%) in VA and 1 (1.3%) had an unknown residence.

**Table 2:** Participant characteristics.

		ARV Treatment Status		
		n (%)		
Characteristic		Naïve	Experienced	p-value
		n = 11	n = 68	
			
Age [IQR]		30.6 [13.3]	48.8 [18.6]	<0.05
Sex at Birth				0.6028
	Male	8 (72.7)	44 (64.7)	
	Female	3 (27.3)	24 (35.3)	
Race				<0.05
	Non-Hispanic Black	7 (63.6)	59 (86.8)	
	Non-Hispanic White	3 (27.3)	2 (2.9)	
	Hispanic	0 (0.0)	5 (7.4)	
	Unknown	1 (9.1)	2 (2.9)	
HIV Risk Category				0.2615
	IDU	1 (9.1)	6 (8.8)	
	MSM	7 (63.6)	20 (29.4)	
	HRH	2 (18.2)	27 (39.7)	
	Unknown	1 (9.1)	14 (20.6)	
	Other	0 (0.0)	1 (1.5)	
Laboratory Results [IQR]				
Median Nadir CD4 (cells/μL)		455 [290.5]	103 [209.8]	<0.05
Median Current CD4 (cells/μL)		455 [419.0]	192 [322.5]	<0.05
Median Viral Load (copies/mL)		5.3x10^4^	2.3x10^4^	0.6199

IDU; Injection Drug User, MSM; Men Who Have Sex with Men, HRH; High Risk Heterosexual. P-values were determined using Mann-Whitney U test for continuous variables and Pearson’s *χ*^2^ test for categorical variables.

### Describing the impact choice in cutoff percentage had on the number of DRMs detected for HIV-1 PR, RT and IN targets

**Fig 2:**
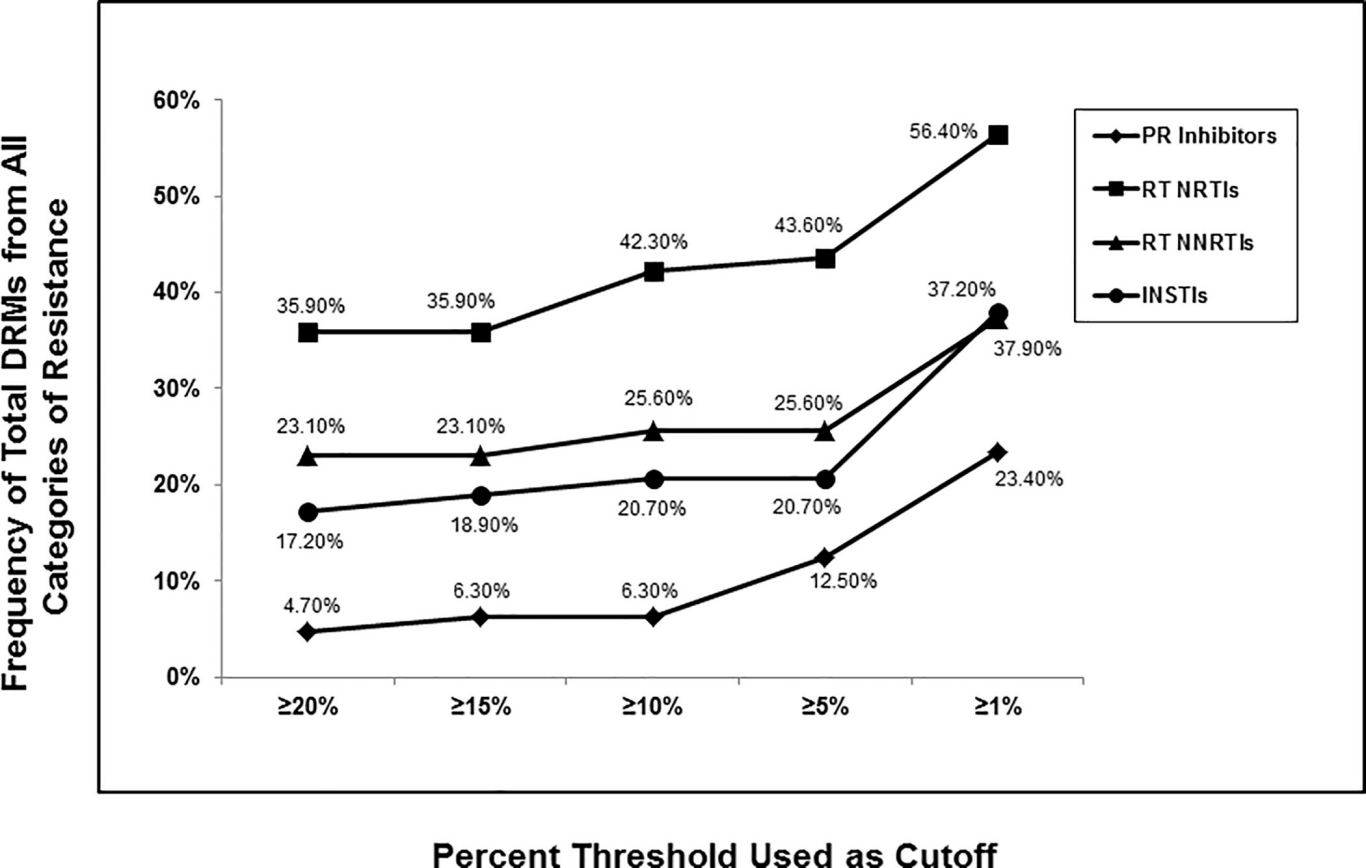
Impact choice in frequency cutoff had on number of total DRMs detected. Frequency of the total numbers of DRMs, including Potential Low-level, Low-level, Intermediate and High-level resistance detected for Protease Inhibitors (PIs) (n = 64), Nucleoside Reverse Transcriptase Inhibitors (NRTIs) and Non-Nucleoside Reverse Transcriptase Inhibitors (NNRTIs) (n = 78) and Integrase Strand Transfer Inhibitors (INSTIs) (n = 58) was determined across 5 different % frequency cutoffs including ≥20%, ≥15%, ≥10%, ≥5% and ≥1% using the Stanford HIVdb program from HyDRA Web data.

One or more amplicons were successfully generated from each of the 79 plasma samples for the following gene targets: PR; 64 (81%), RT; 78 (99%) and IN; 58 (73%). The success rate to generate these 3 amplicons in the group of 11 drug-naïve participants was similar to that of the entire group: PR; 10/11 (91%), RT; 11/11 (100%) and IN; 7/11 (64%). Fig 2 illustrates frequencies of the total number of DRMs detected across all four categories, including: Potential Low-level, Low-level, Intermediate and High-level resistance, for the four different drug classes at 5 different cutoff percentages used (≥20%, ≥15%, ≥10%, ≥5% and ≥1%). In each drug class analyzed, DRM frequencies increased as the cutoff percentage decreased. Highest rates of DRMs were seen for the NRTIs (35.90–56.40%) followed by NNRTIs (23.10–37.20%), INSTIs (17.20–37.90%) and PIs (4.70–23.40%).

Of 11 drug-naïve participants, DRMs were detected in 8 (72.7%). However, only 3 (27.3%) participants had DRMs present at a frequency ≥5%. These included two participants with the INSTI-mutation E157Q at frequencies of 19.67% or 91.49% (inferring potentially Low-level resistance for EVG and RAL), and one participant with the RT-mutation T215C at 5.89% (inferring Low-level resistance for AZT and DDI).

**Fig 3:**
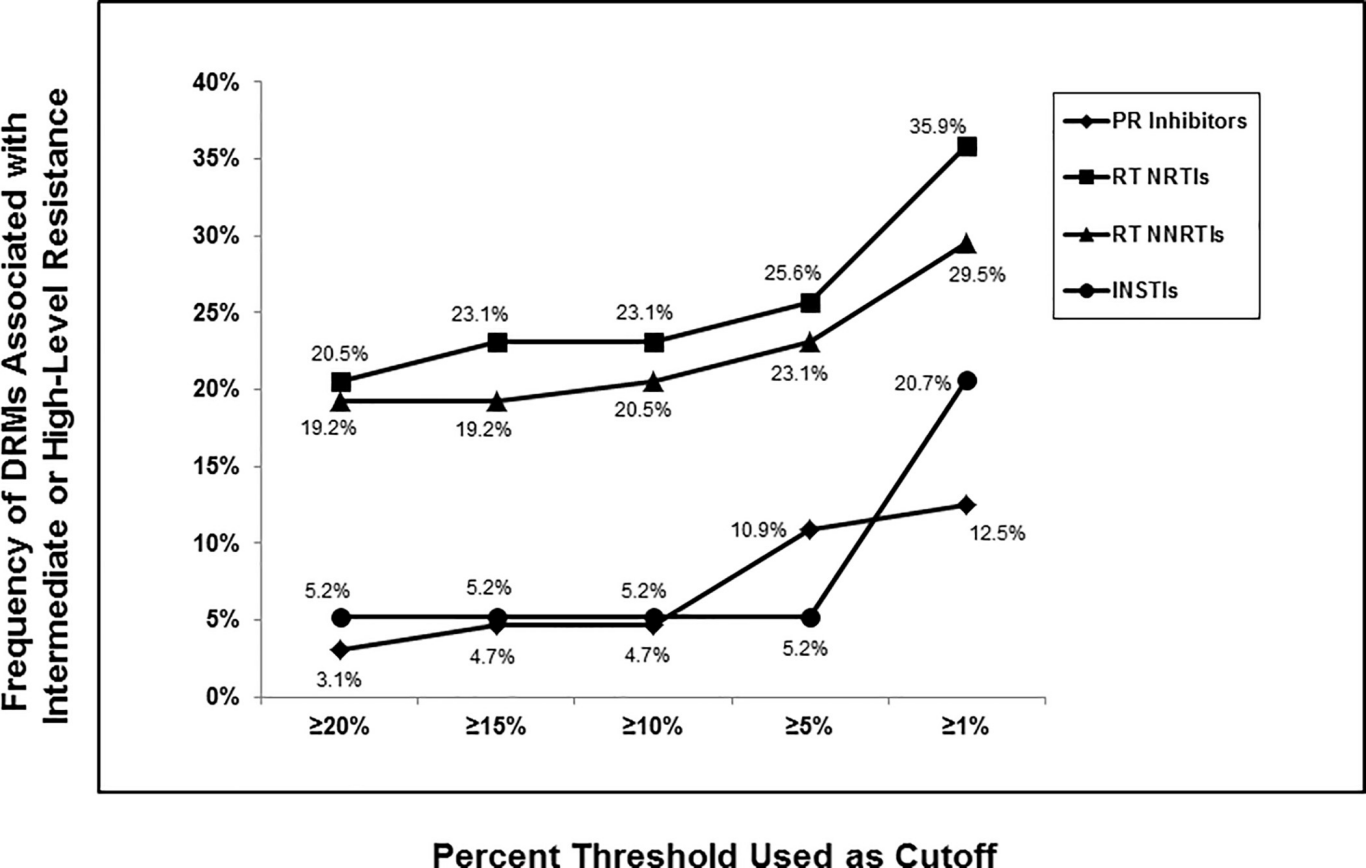
Impact choice in frequency cutoff had on number of DRMs resulting in intermediate and/or high-level resistance. Frequency of the numbers of DRMs, including Intermediate and High-level resistance, that were detected for Protease Inhibitors (PIs) (n = 64), Nucleoside Reverse Transcriptase Inhibitors (NRTIs) and Non-Nucleoside Reverse Transcriptase Inhibitors (NNRTIs) (n = 78) and Integrase Strand Transfer Inhibitors (INSTIs) (n = 58) was determined across 5 different % frequency cutoffs including ≥20%, ≥15%, ≥10%, ≥5% and ≥1% using the Stanford HIVdb program from HyDRA Web data.

Fig 3 illustrates frequencies of DRMs resulting only in Intermediate or High-Level Resistance for the four different drug classes at the same 5 cutoff percentages. Comparing these data to that seen in Fig 2, the highest rates of DRMs were again seen for the NRTIs (20.5–35.9%) followed by NNRTIs (19.2–29.5%), INSTIs (5.2–20.7%) and PIs (3.1–12.5%). The differences in DRM rates for IN and PR were not as dramatic when focusing solely on those DRMs resulting in Intermediate or High-Level resistance compared to the differences seen for total DRMs.

Of all the IN and PR DRMs detected in this study, most (61%; PR and 56%; IN) were present <5%. Only 1 participant had DRMs resulting in High-level resistance to all four INSTIs and these were all present at frequencies >15%. These INSTI-mutations included T97A, E138A/K, Q143H, G140S and V151I. Importantly, the most common INSTI-mutation by far was E157Q; consistently present >20% (median frequency, 84.1%), but this DRM has little effect on INSTI susceptibility.

### DRMs detected using HyDRA Web were compared to those generated using SmartGene and/or PASeq Web

**Fig 4:**
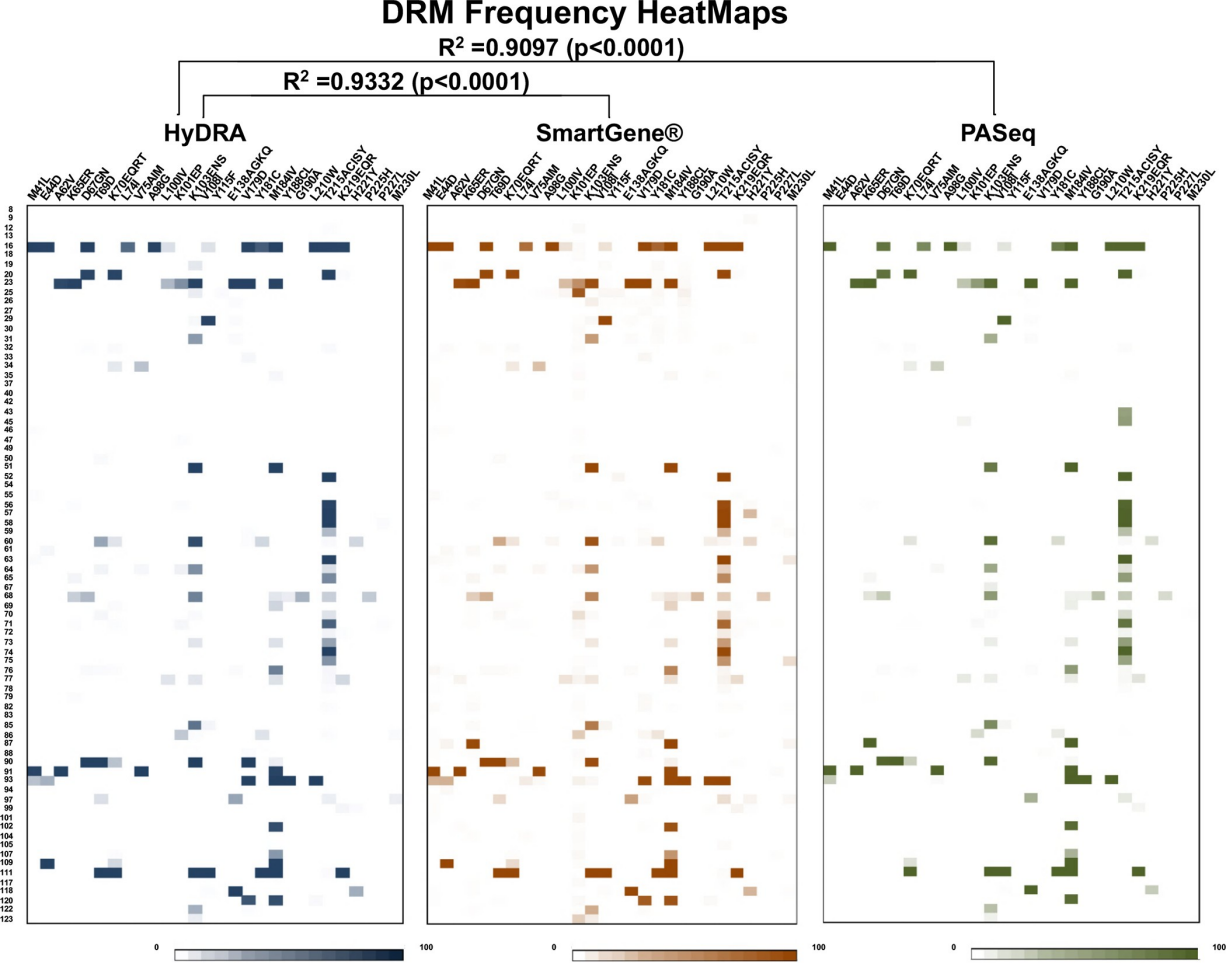
RT DRM frequency data represented as HeatMaps from three different pipelines. Using Microsoft Excel conditional formatting function was applied to generate a color gradient from white (0% DRM) to darkest color (100% DRM). The pipelines used included HyDRA Web (blue colored scheme), SmartGene (brown colored scheme) and PASeq Web (green colored scheme). Above each of the 3 Heatmaps is the key to amino acid changes. The numbers located on the far-left hand side of the figure represents specimen study numbers listed in numerical order.

Fig 4 illustrates the three HeatMaps generated from 78 participants’ RT sequences. These maps provide a DRM snap shot for a single participant (e.g., from no DRMs detected for participant #8 to as many as 13 DRMs detected for participant #16), as well as the most common mutations occurring among the group (RT-mutation T215ACISY; n = 19, RT-mutation M184IV; n = 17 and RT-mutation K103ENS; n = 14).

The Mantel test was used to calculate correlations between HyDRA Web and SmartGene matrices, as well as between HyDRA Web and PASeq Web for detecting DRMs from RT sequences. The R^2^ (coefficient of determination) calculated and corresponding p-values were 0.9332 (p<0.0001) and 0.9097 (p<0.0001), respectively. In both comparisons, we rejected the null hypothesis that distances among objects between our three matrices were not linearly correlated and accepted the alternative hypothesis that the three distance matrices were highly correlated.

The Mantel test was again used to calculate correlations between HyDRA Web and PASeq Web matrices (HeatMaps not shown) for detecting DRMs from PR and IN sequences. The R^2^ (coefficient of determination) calculated and corresponding p-values were 0.9993 (p<0.0001) and 0.9765 (p<0.0001), respectively.

Of all the IN and PR DRMs detected in this study, most (61%; PR and 56%; IN) were present <5%. Only 1 participant had DRMs resulting in High-level resistance to all four INSTIs and these were all present at frequencies >15%. These INSTI-mutations included T97A, E138A/K, Q143H, G140S and V151I. Importantly, the most common INSTI-mutation by far was E157Q; consistently present >20% (median frequency, 84.1%), but this DRM has little effect on INSTI susceptibility.

### Comparing DRM interpretation reports generated using Stanford HIVdb program with those generated using SmartGene Web-based software

DRM interpretation reports were generated to infer ART efficacy for the 7 NRTIs (ABC, AZT, D4T, DDI, FTC, 3TC and TDF) and 4 NNRTIs (EFV, ETR, NVP and RPV) for the pipeline described here and for the SmartGene pipeline, as both pipelines used the same 5 interpretation categories: Susceptible, Potential Low-level Resistance, Low-level Resistance, Intermediate-level Resistance and High-level Resistance. In all, there were 546 comparisons made for the 78 RT sequences across 7 NRTIs, and 312 comparisons for those 78 RT sequences across 4 NNRTIs. There was perfect agreement in 95.1% (519/546) of interpretations generated for NRTIs and in 98.1% (306/312) for NNRTIs. The *r*_*s*_ value calculated using the Spearman’s Correlation was 0.97757 (p<0.0001), showing a very strong positive agreement between the two data sets. DRM interpretation reports generated using the HyDRA Web pipeline could not be compared against PASeq Web because the interpretation categories used for the latter pipeline combine Susceptible with Potential Low-level resistance, and combine Low-level resistance with Intermediate resistance when generating DRM Interpretation reports.

### Transmission clusters generated using HIV-TRACE

**Fig 5:**
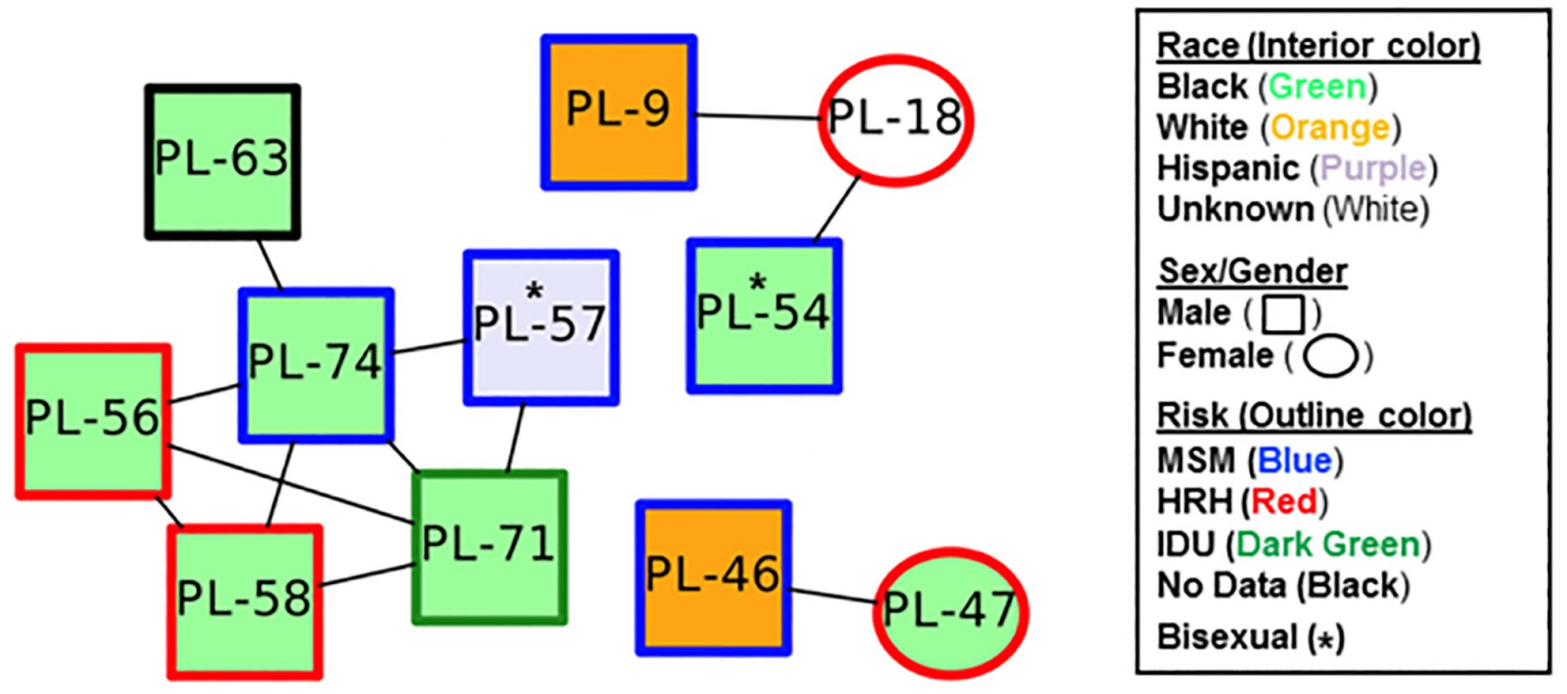
Transmission clusters were created using HIV-TRACE software based on RT NGS sequences generated using the ≥20% frequency cutoff along with participants’ race, sex/gender and HIV risk categories.

The 78 RT consensus sequences along with demographic data were used to look for evidence of relatedness using HIV-TRACE software. Eleven of 78 (14.1%) participants were part of a transmission cluster (Fig 5); represented as a dyad, triad and 6-person cluster. Nine of 11 individuals (81.8%) were male; five of whom had the HIV risk category of MSM, and two who were bisexual. The remaining two participants were HRH females with the HIV risk category of High-Risk Heterosexuals (HRH). All 3 clusters contained HIV risk behavior categories of MSM and HRH. Ten of 11 participants found within these 3 clusters were treatment-experienced; only 1 (PL-46) was treatment-naïve.

Common DRMs were found within a cluster. In the dyad, both participants had the INST-mutation E157Q at 91.49% and 84.07% for PL-46 and PL-47, respectively. This is a polymorphic mutation having little effect on INSTI susceptibility [17,18]. The entire 6-person cluster had the RT-mutation T215C at 98.33%, 99.57%, 99.64%, 98.93%, 82.62% and 99.18% in PL-56, PL-57, PL-58, PL-63, PL-71 and PL-74, respectively. The RT-mutation T215C is a revertant arising from virus which had the RT-mutation T215Y/F and does not reduce NRTI susceptibilities. Two participants (PL-56 and PL-71) within the 6-member cluster also had the RT-mutation K65E at a frequency of 1.06% and 1.05%, respectively and the RT-mutation K101E at a frequency of 1.6% and 3.45%, respectively. The triad cluster had no DRMs detected.

## Discussion

In this study, we demonstrated excellent correlation between the HyDRA Web pipeline, a free, publicly-available software used to analyze NGS data for detecting DRMs and generating DRM interpretation reports, and one or more pipelines described here. Importantly, lab personnel without extensive bioinformatics expertise were able to perform the analyses. Using this approach could provide a tool to assess population-based DRMs in conjunction with specific anti-retroviral drugs to create an antiviragram to help monitor DRMs seen within a community.

The approach using HyDRA Web to analyze MiSeq NGS data compared favorably to SmartGene and to PASeq, another publicly-available pipeline. These data were similar to those recently published by Perrier et al. comparing SmartGene to Geneious, another commercially-available software where the R^2^ = 0.995 using MiSeq-generated HIV-1 RT sequences [4].

Arias et al. also found strong agreement with MiSeq-generated HIV-1 NGS data analyzed by HyDRA Web software using a 20% frequency cutoff to that using Sanger-based sequencing; additional DRMs were seen from the NGS data when using a 5% frequency cutoff [19]. In addition, Tzou et al. reported a strong correlation between data generated using Sanger sequencing and that from an *in vitro* diagnostic NGS assay [20].

All publicly-available software used in NGS analyses have their own strengths and limitations. Advantages to HyDRA software include its user-friendly interface with customizable analysis settings for DRM frequency (default is >1%). We found we could upload and analyze more sequences at one time on HyDRA’s website than was possible with some of the other free software programs we tried. However, unlike PASeq (https://paseq.org), HyDRA cannot generate clinical interpretation reports without first entering that data manually into the Stanford HIVdb program.

In a recent 2018 publication by the Centers for Disease Control and Prevention (CDC), HIV-TRACE was included as a bioinformatics tool for Health Departments to use when analyzing nucleotide sequences from the *pol* locus to identify clusters representing transmissions [21]. This software would be helpful for local health departments to adopt so as to help focus their surveillance efforts on the larger clusters found in their communities. Our analysis using HIV-TRACE included RT sequences only, but in the future, with a larger data set, we plan to include PR and IN sequences as well.

Time to actionable results, from obtaining specimens to completing NGS data analysis using this workflow described here, is ~7 to 9 days; a near-real timeframe to assist public health surveillance efforts underway in Washington, DC to combat the HIV epidemic; a goal similar to that described by Poon et al. for British Columbia, Canada [22].

When using NGS, it is important to differentiate natural polymorphisms at resistance sites from mutations conferring phenotypic drug resistance. The high frequency of E157Q may not be a resistance mutation per se. Rather, it may reflect a natural polymorphism with favored fitness or transmissibility [17, 18, 23, 24]. With more labs switching to NGS platforms, the question around relevancy of low frequency DRMs remains. Kyeyune F. et al. describes how presence of minor variant DRMs in a treatment-naïve population predicts increased rates of future virologic failure [3]. While in a longitudinal study, Moscona et al. reports that low frequency variants do not become high frequency variants [25]. Guidance around which DRM frequency cutoff to select for data analysis to provide clinically-relevant interpretations of NGS data is urgently needed and remains a work in progress [26]. However, several recent studies now suggest a 5% threshold is clinically-relevant for determining DRMs from NGS data [20, 27, 28]. There is a risk-benefit issue to consider when establishing this cutoff. If many minor variants are detected and reported, the result may be that no ART regimen appears viable, which is discouraging and likely untrue. Alternatively, healthcare providers could begin to ignore these DRM reports.

Limitations of this study include our inability to produce all 3 amplicons 100% of the time for each participant. This is due in part to the natural challenges of high mutation rates in HIV-1. We have since designed additional primer pairs not only to improve our amplicon success rate but to include amino acids #236, 238, 318 and 348 found at the 3’ end of RT gene. Success in generating NGS data from participants with viral loads <1,500 copies/mL will require collecting larger blood volumes or including a virus concentration step during sample preparation. Sequencing virus from plasma with low viral load may not be clinically relevant for risk of transmission [29], but may be an important public health tool for better understanding transmission networks. Lastly, because we incorporated Nextera XT DNA Library Prep chemistry, a process that fragments DNA, we were unable to determine whether multiple DRMs were present on single HIV-1 genomes. Thus, our ARV clinical interpretations represent a maximal estimation of impact of DRMs present in participant’s plasma.

The question of accuracy around NGS data is an important one. The average MiSeq error rate for these 9 runs was calculated to be 0.54%. However, it should be noted that the actual MiSeq error rates will be lower than that estimated for single strand sequencing because paired-end sequencing was performed here [30]. The error rate for the high fidelity *Taq polymerase* used in these experiments is 0.009%, as stated by the manufacturer (Takara). Even with a high fidelity Taq polymerase, errors do occur during PCR amplification, so using a technology like Primer ID can help to correct for this type of error [31]. Overall, the quality of our NGS runs was high; the average ≥Q30 score from these 9 MiSeq NGS runs was 92.80 ± 2.02 (SD).

Several parallels can be drawn from our current NGS data and that of Sanger-generated sequences collected between 1999–2014 from this DC Cohort [32, 33]. Highest levels of DRMs continue to be seen for RTIs, with the RT-mutations K103N, M184V and T215Y mutations being most common in both cases. We again found evidence for transmitted INSTI DRMs among treatment-naïve participants. It must be noted that the present study represents a limited data set of 79 participants, so it will be important to expand our sample size.

In summary, we have described and validated a comprehensive, easy-to-use and affordable NGS approach for tracking HIV-1 drug resistance and identifying potential transmission clusters within the community. Our goal would be to make this NGS approach available for others interested in transitioning to an NGS platform for their public health surveillance efforts.
